# Potential Role of Methotrexate Polyglutamates in Therapeutic Drug Monitoring for Pediatric Inflammatory Bowel Disease

**DOI:** 10.3390/ph14050463

**Published:** 2021-05-14

**Authors:** Ryan Morrow, Ryan Funk, Mara Becker, Ashley Sherman, Leon Van Haandel, Taina Hudson, Rebecca Casini, Valentina Shakhnovich

**Affiliations:** 1 Children’s Mercy Kansas City, Kansas City, MO 64108, USA; aksherman@cmh.edu (A.S.); thudson@cmh.edu (T.H.); vshakhnovich@cmh.edu (V.S.); 2 University of Missouri Kansas City School of Medicine, Kansas City, MO 64108, USA; 3 Department of Pharmacy Practice, University of Kansas, Kansas City, KS 60047, USA; ryanfunk@kumc.edu; 4 Department of Pediatrics, Duke University Hospital, Durham, NC 27705, USA; mara.becker@duke.edu; 5 Abbvie Pharmaceuticals, North Chicago, IL 60085, USA; leon_v_haandel@hotmail.com; 6 NorthShore University Health System, Skokie, IL 60201, USA; rebecca.a.casini@gmail.com

**Keywords:** methotrexate, methotrexate polyglutamates, infliximab, inflammatory bowel disease, Crohn’s disease, ulcerative colitis, pediatric

## Abstract

Inside cells, the immunomodulator methotrexate (MTX) undergoes the addition of glutamates to form methotrexate polyglutamates (MTX-Glu)—promising biomarkers of systemic exposure and treatment response to MTX in rheumatology. MTX-Glu are underexplored in Inflammatory Bowel Disease (IBD), with no data in pediatrics. In this cross-sectional secondary analysis, we assessed the relationships between MTX-Glu and MTX dose and treatment response in pediatric IBD. Twenty-one children with IBD, receiving maintenance therapy with infliximab (IFX) and MTX, had MTX-Glu_1–6_ concentrations and IFX troughs/antibodies measured and disease activity assessed for comparison in remission vs. active IBD using non-parametric tests, with associations explored using Spearman’s correlation (ρ) and regression analyses; SASv9.4 (α = 0.05). Total and long-chain MTX-Glu correlated with MTX dose (ρ = 0.51 and 0.56, respectively; *p* ≤ 0.02). In children with Crohn’s disease (*n* = 19), short-chain MTX-Glu_1–2_ were 2.5-fold higher in remission vs. active disease, approaching statistical significance (*p* = 0.066), with no statistical differences in IFX trough (*p* = 0.549) between groups. Our study highlights a potential role for long-chain MTX-Glu in the therapeutic drug monitoring of MTX in IBD. It is the first study in pediatric IBD and, although statistical significance was not reached, our findings also suggest that higher short-chain MTX-Glu levels may be associated with IBD treatment response to MTX in children.

## 1. Introduction

Methotrexate (MTX) is a folic acid analog used as monotherapy, or in combination with anti-TNF-α agents, for the treatment of autoimmune disorders [1]. To exert its action, MTX enters cells via folate transporters and receptors, and undergoes the addition of glutamic acid (Glu) residues by folylpolyglutamate synthetase to form methotrexate polyglutamates (MTX-Glu_n_) [2], where *n* represents the number of glutamic acid residues, with MTX-Glu_1_ denoting the parent form of MTX. The addition of glutamic acid to MTX occurs sequentially, and the metabolites formed are classified based on the length of the glutamate side chain, where glutamate Glu_1 and 2_ are considered short-chain and Glu_3–6_ long-chain [3]. Unlike MTX-Glu_1–2_, MTX-Glu_3–6_ are not effluxed from the cell efficiently and, therefore, reflect steady-state intracellular methotrexate concentration. In addition, the polyglutamated metabolites of MTX have a differential activity profile compared to the parent drug, with similar inhibitory activity against dihydrofolate reductase, but increased direct inhibitory activity against folate-dependent enzymes involved in purine and pyrimidine biosynthesis [4]. In rheumatology, erythrocyte concentrations of MTX-Glu_n_ have been associated with systemic exposure to MTX and MTX treatment efficacy [3,5,6,7]. Data are lacking in inflammatory bowel disease (IBD), where erythrocyte MTX-Glu_n_ levels could offer a clinically useful biomarker for MTX responsiveness and/or a measure for therapeutic drug monitoring—currently unavailable for this drug despite its frequent use. The aim of this prospective, single-center, cross-sectional, secondary analysis of pediatric patients receiving stable doses of infliximab (IFX) and MTX for IBD was to explore the relationships between MTX-Glu_n_ and MTX dose and treatment response in IBD.

## 2. Results

**Figure 1:**
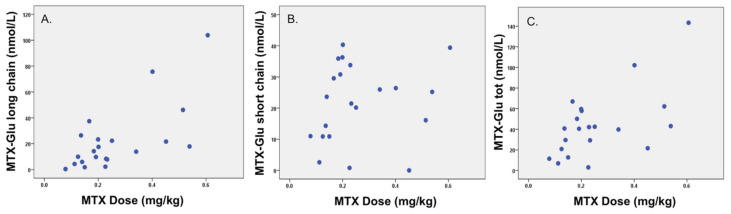
Spearman’s correlation (ρ) between methotrexate polyglutamate (MTX-Glu) concentrations and methotrexate (MTX) dose for long-chain MTX-Glu_3–5_, ρ = 0.56; *p* = 0.009 (**A**); short-chain Glu_1–2_, ρ = 0.27; *p* = 0.244 (**B**); and total MTX-Glu (MTX-Glu_tot_), ρ = 0.51; *p* = 0.018 (**C**).

Children included in this secondary analysis completed a convenience-sampling cross-sectional investigation of IFX pharmacokinetics across different pediatric autoimmunity diagnoses [8]. Of the 21 children enrolled (median age 16 years (IQR 12, 17), 38% F, 90% Crohn’s disease), 10 had active disease (six mild, three moderate, and one severe) and 11 were in remission (Paris Disease Classification in Table 1). One child with active disease (mild), and undetectable IFX trough, had anti-IFX antibodies. The two study groups were otherwise comparable in age, at 16.0 (13.0, 17.0) vs. 14.5 (9.0, 19.0) years, and disease duration, at 2.5 (1.8, 5.2) vs. 3.8 (1.1, 4.5) years; both *p* ≥ 0.8. Clinically prescribed MTX doses for patients varied from 5 to 25 mg weekly, with 86% oral administration. To account for inter-individual variability in MTX dose, as well as variability in patient age and size in our pediatric cohort (5–21-year-olds), the MTX dose was adjusted for total body weight (mg/kg). Adjusted for weight (mg/kg), the MTX dose correlated significantly with long-chain MTX-Glu_3–5_ (ρ = 0.56; *p* = 0.009) and MTX-Glu_Total_ (ρ = 0.51; *p* = 0.018), but not short-chain Glu_1–2_ (ρ = 0.27, *p* = 0.244); see Figure 1. MTX-Glu_6_ was undetectable across the study population.

**Figure 2:**
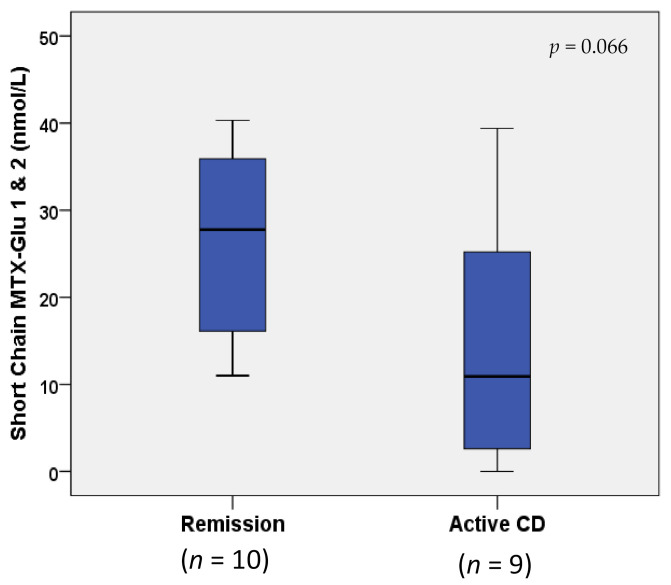
Box and whisker plot comparison of erythrocyte MTX-Glu_1–2_ concentrations in remission vs. active Crohn’s disease. Horizontal line in boxes represents median value, with whiskers representing the range.

MTX-Glu_n_ concentrations were not statistically different between children with IBD in remission vs. active disease (*p* > 0.1; data not shown). However, when MTX-Glu_n_ concentrations were compared only in children with Crohn’s disease (*n* = 10 remission vs. *n* = 9 active), an increase greater than two-fold in short-chain MTX-Glu_1–2_ concentrations (nmol/L) was observed in remission (27.77 (16.10, 35.90)) vs. active Crohn’s disease (10.90 (2.60, 25.20)), approaching statistical significance (*p* = 0.066; Figure 2). The two Crohn’s disease study groups were receiving comparable dosing of both MTX (0.20 (0.17, 0.23) vs. 0.23 (0.15, 0.45) mg/kg weekly) and IFX (9.24 (7.45, 10.04) mg/kg every 4.0 (4.0, 6.0) weeks vs. 9.56 (8.49, 10.03) mg/kg every 5.0 (4.0, 6.0) weeks); *p* ≥ 0.554 (Table 2). Median and IQR IFX troughs (μg/mL) were within therapeutic range (i.e., ≥3–7 μg/mL [9]) in both groups, and not statistically different between groups (25.28 (5.35, 38.96) vs. 15.54 (7.11, 24.15)); *p* = 0.540 (Table 2). However, when IFX was included as a covariate in a logistic regression model, the differences in short-chain MTX-Glu_n_ between remission and active Crohn’s disease became less apparent (*p* = 0.130).

Cut-off values for MTX-Glu_n_ concentrations previously associated with treatment response in rheumatology [5] were probed, and none were found to discriminate remission from active disease in IBD or in the IBD subset with Crohn’s disease only (data not shown).

## 3. Discussion

To the best of our knowledge, this is the first study of MTX-Glu_n_ levels in pediatric IBD and it offers valuable insights for future investigations. First, our data demonstrate that long-chain MTX-Glu_n_ levels correlate with methotrexate dose and could, therefore, serve as markers of systemic MTX exposure, offering a therapeutic drug monitoring option for MTX. Second, in children with Crohn’s disease, short-chain MTX-Glu_n_ levels were over two-fold higher in patients with disease in remission, compared to active disease, suggesting that short-chain MTX-Glu may have a role in Crohn’s disease treatment responsiveness to MTX.

Although our observation of higher short-chain MTX-Glu levels in CD in remission compared to active CD did not reach statistical significance (*p* = 0.066), this is likely due to limitations in the sample size and further obscured by participants receiving concomitant IFX therapy, as suggested by our multivariable regression analysis. Nevertheless, the observed trend in short-chain MTX-Glu suggests that efficient intracellular drug uptake and/or primary intracellular conversion of MTX-Glu_1_ to short-chain MTX-Glu_2_ may be associated with the treatment responsiveness of pediatric Crohn’s disease to MTX. An alternative explanation for this observation is that primary polyglutamation may be impaired in active disease, perhaps through altered intracellular drug uptake or inflammatory cytokine-mediated modulation of enzymes in the polyglutamation pathway (e.g., folypolyglutamate synthase or gamma glutamyl hydrolase, responsible for glutamation and deglutamation, respectively) [10]. Future studies should be aimed at understanding such sources and implications of inter-individual variability in polyglutamation for the treatment of pediatric IBD, where equipoise remains regarding up-front identification of patients who benefit most from MTX therapy (alone or in combination with biologics).

Ours is the first study of MTX-Glu_n_ in pediatric IBD and our findings regarding long-chain MTX-Glu_n_ echo findings in juvenile idiopathic arthritis (JIA) and rheumatoid arthritis (RA)—two chronic, autoimmune, inflammatory conditions also treated with MTX. Becker et al. previously demonstrated increased MTX-Glu_3–5_ formation as a function of MTX dose, route of administration, and duration of drug exposure in patients with JIA. Specifically, MTX dose escalation resulted in a preferential increase in long-chain MTX-Glu_n_, at the expense of short-chain MTX-Glu_n_ [11]. A study in RA also demonstrated a positive association of MTX dose with long-chain MTX-Glu_3_, MTX-Glu_4_, MTX-Glu_5_, and MTX-Glu_3–5_, while observing a weaker association between dose and total MTX-Glu_1–5_ [12], analogous to the observations in our study.

Previous studies in rheumatology have also commented on the relationship between MTX-Glu_n_ and treatment response [3,5,6,7]. No statistically significant differences in individual or total MTX-Glu_n_ concentrations were observed in children with IBD in remission vs. active disease in our study; however, MTX therapy is felt to be less effective for ulcerative colitis than Crohn’s disease [13,14]. Looking only at children with Crohn’s disease (*n* = 19), a positive trend toward higher short-chain MTX-Glu_n_ concentrations was observed in children with remission vs. active disease, approaching statistical significance (*p* = 0.066). The two Crohn’s disease study groups were otherwise comparable for age and disease duration, without statistically significant differences in IFX troughs between groups. Although a trend toward greater IFX troughs (both absolute and adjusted for clinical variability in IFX dose and interval) was observed in children with remission vs. active Crohn’s disease, this trend did not approach statistical significance (*p* = 0.549), and mean and median IFX troughs for both groups fell well within the therapeutic range (i.e., ≥3–7 μg/mL [9]); see Table 2. Nevertheless, we acknowledge that concomitant IFX therapy may be a confounding variable in our MTX-Glu analyses, as supported by observations of a less obvious difference in short-chain MTX-Glu_n_ between Crohn’s disease in remission and active disease after incorporating IFX into a multivariable logistic regression model. While some previous studies of IFX and MTX have shown a questionable benefit of combination therapy for Crohn’s disease [15], our findings raise the possibility that the benefit of combination therapy may be related to the intracellular MTX-Glu_n_ concentration achieved, emphasizing the need for further investigation of MTX-Glu_n_ monitoring in IBD.

Although published rheumatology data to date are felt insufficient to recommend the definite implementation of MTX-Glu_n_ therapeutic drug monitoring in clinical practice [16], a systematic review of the rheumatology literature from 2015 provides supportive evidence for the role of MTX-Glu_n_ as biomarkers of autoimmune disease response to MTX treatment [7]. In a study of MTX-naïve patients with RA, short-chain MTX-Glu_2_ correlated with an improvement in clinical assessments over 16 weeks of MTX therapy. [3] In another study of RA, de Rotte et al. proposed concentrations of MTX-Glu_2_ > 22 nmol/L and MTX-Glu_Total_ > 74 nmol/L as predictors of moderate/good clinical response to MTX. [5] Cut-off values to discriminate MTX responders from non-responders could not be established in our study, but this is likely secondary to limitations in sample size.

Previous studies of MTX-Glu_n_ in IBD are sparse and limited to adult observational studies smaller than ours, which may explain why prior studies also failed to demonstrate a statistically significant association between MTX-Glu_n_ and IBD treatment response. [17,18]. In a cross-sectional study of 12 adults, Fischer et al. observed higher long-chain MTX-Glu_3–5_ in patients with Crohn’s disease in remission vs. active disease, but the trend did not reach statistical significance. [17] Brooks et al. found long-chain MTX-Glu_4–5_ to correlate inversely with MTX efficacy in 18 adults with Crohn’s disease, with higher levels noted in patients experiencing gastrointestinal adverse effects [18]. However, findings of an inverse relationship between MTX-Glu_n_ and MTX efficacy are not substantiated in most rheumatology literature [5,6,7], except for one study that found a positive correlation for MTX-Glu_n_ with MTX dose, but a negative correlation with treatment outcomes in RA [19].

Studies of MTX-Glu_n_ in children are rare and, to the best of our knowledge, ours is the first pediatric study in IBD. In a pediatric study of JIA, higher concentrations of long-chain MTX-Glu_n_ (MTX-Glu_3–5_) were associated with lower disease activity at 3 months and 1 year of MTX therapy. [6] The cross-sectional nature of our pediatric study does not allow for a longitudinal assessment. However, despite the significant positive correlation of long-chain MTX-Glu_n_ with MTX dose in our study (ρ = 0.56; *p* = 0.01), we did not observe a relationship with disease response, suggesting that the relationship between long-chain MTX-Glu_n_ and treatment response may be disease-specific and could differ between JIA and pediatric IBD.

Although this is the largest study of MTX-Glu_n_ in IBD to date, the limitations of our study reside with its small sample size and cross-sectional design. The present study design does not allow us to comment on the relationship between maintenance MTX-Glu_n_ concentrations and long-term disease outcomes in IBD. The decision to study patients receiving combination therapy with MTX and IFX may be viewed as another study limitation; however, at our institution, MTX is most often used in combination with biologics, rather than as monotherapy, for the treatment of IBD. Thus, the selected study population is more generalizable to the patient population at large, which is important for translating study results into clinical practice. To account for potential confounders from concomitant IFX treatment, IFX troughs were measured at the time of MTX-Glu_n_ assessment, using a single CLIA-approved assay, and incorporated into our statistical analyses. Future investigations should be aimed at longitudinal, combination, and monotherapy MTX studies, to build on our observations of the potential role of long-chain MTX-Glu_n_ in the therapeutic drug monitoring of systemic exposure to MTX, and short-chain MTX-Glu_n_ as potential biomarkers of IBD responsiveness to MTX treatment. Prospective, multicenter, dose-controlled trials of MTX could help to identify therapeutic target ranges for MTX-Glu_n_ in IBD.

## 4. Materials and Methods

**Figure 3:**
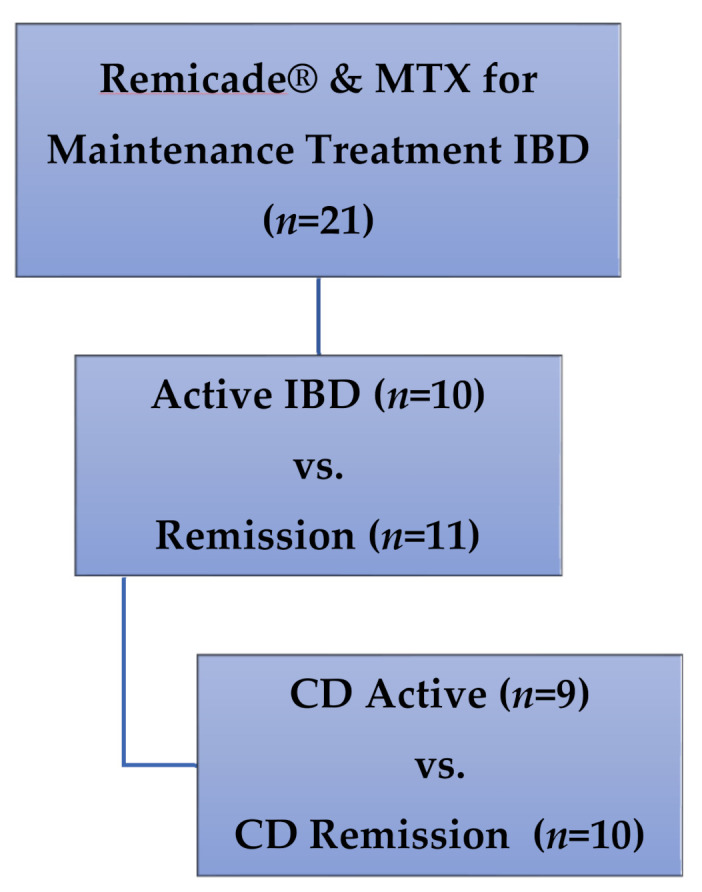
Patients included in this secondary analysis of methotrexate polyglutamates in pediatric IBD. MTX: methotrexate, IBD: inflammatory bowel disease, CD: Crohn’s disease.

Patients: Children enrolled in a cross-sectional study of IFX monotherapy vs. combination IFX therapy with concurrent immunomodulators, and receiving medication doses in accordance with prescriber preference for clinical care of IBD [20], were included in this secondary analysis. All participants were enrolled at a single outpatient infusion center at the Children’s Mercy Hospital (Kansas City, MO, USA). Only patients receiving therapy with both IFX (Remicade^®^) and MTX were included in this secondary analysis (Figure 3), with maintenance dosing defined as no changes in dose or interval of either drug for at least two IFX infusion cycles.

Clinical parameters: Clinical parameters were assessed using Physician Global Assessment (PGA) scores via agreement by two independent pediatric gastroenterologists. Due to limitations in sample size, PGA mild, moderate, and severe were grouped together as active disease (active) and compared to quiescent disease (remission). 

Analytical techniques and validation: MTX-Glu_1–6_ concentrations were measured in erythrocytes, using high-performance liquid chromatography/tandem mass spectrometry utilizing a previously established assay [20]. IFX troughs and anti-IFX antibodies (anti-IFX) were measured using a CLIA-approved NF-kB luciferase gene-reporter assay, with lower and upper limits of quantification at 0.65 and 40 μg/mL, respectively (ARUP Laboratories, Salt Lake City, Utah, UT, USA).

Statistical analysis: Nonparametric tests (e.g., Wilcoxon Rank Sum and Fisher’s Exact tests) were used to compare MTX-Glu, IFX, anti-IFX, laboratory, and demographic data in children with remission vs. active disease. Spearman’s correlation (ρ) was used to look for relationships between continuous variables, and multivariable logistic regression models were used to look for differences between study groups, while controlling for covariates. Previously reported cut-off values in rheumatology of MTX-Glu_Total_ > 74 nmol/L and MTX-Glu_2_ > 22 nmol/L were probed to discriminate remission from active disease in IBD [5]. A significance level of 0.05 in SAS, version 9.4 (SAS Institute Inc., Cary, NC, USA), was used for all analyses. Unless otherwise specified, data are reported as median (IQR).

## Figures and Tables

**Table 1:** Paris Classification. Age at diagnosis: A1a (0–<10 years); A1b (10–<17 years). Location: L1: distal 1/3 ileum +/− limited cecal disease; L2: colonic; L3: ileocolonic; L4a: upper disease proximal to ligament of Treitz; L4b: upper disease distal to ligament of Treitz and proximal to distal 1/3 ileum. Extent: E1: ulcerative proctitis; E2: Left-sided UC (distal to splenic flexure); E3: extensive (hepatic flexure distally); E4: pancolitis (proximal to hepatic flexure). Behavior: B1: nonstricturing nonpenetrating; B2: structuring; B3: penetrating; B2B3: both penetrating and stricturing disease, either at the same or different times; *p*: perianal disease modifier. Severity: S0: never severe; S1: ever severe. Growth: G0: no evidence of growth delay; G1: growth delay.

Remission (*n* = 11)	A1a 36%	L1 25%	B1 50%	G0 100%
		L2 0%	B2 0%	
		L3 50%	B3p 50%	G1 0%
		L4b 25%	S1 0%	
	A1b 64%	L1 17%	B1 57%	G0 43%
		L2L4a 17%	B2B3 14%	
		L3 33%		G1 43%
		L3L4aL4b 17%	B3 14%	Missing Data 14%
		L4aL4b 17%		
		E2 17%	S1 14%	
Active (*n* = 10)	A1a 40%	L1L4a 25%	B1 50%	G0 50%
		L3 25%	B2 25%	G1 25%
		L3L4aL4b 25%	B3 0%	Missing Data 25%
		E4 25%	S1 25%	
	A1b 60%	L1 33%	B1 66%	G0 100%
		L2 17%	B2B3p 17%	
		L3L4a 33%	B3 17%	G1 0%
		L3L4aL4b 17%	S1 0%	

**Table 2:** Patient characteristics for children with Crohn’s disease: IFX: infliximab, MTX: methotrexate, _adj_IFX: dose or concentration adjusted for mg/kg IFX received and time since drug administration. All patients received weekly MTX.

	Remission (*n* = 10)	Active (*n* = 9)	
	Median [IQR]	Median [IQR]	*p*-Value
Age (years)	16.0 [13.0, 17.0]	17.0 [11.0, 19.0]	0.902
Weight (kg)	54.6 [50.2, 75.1]	52.3 [39.9, 80.1]	0.653
Hemoglobin (g/dL)	13.0 [12.1, 13.8]	12.2 [11.3, 13.8]	0.438
White Blood Cell Count (×10^3^/mcL)	5.8 [5.0, 6.7]	7.4 [5.0, 10.1]	0.347
Platelet Count (×10^3^/mcL)	264 [237, 299]	325 [243, 394]	0.713
ESR (mm/h)	8.0 [6.0, 10.0]	41.0 [18.0, 42.0]	0.006
CRP (mg/dL)	0.5 [0.5, 0.8]	1.4 [0.8, 2.2]	0.028
Albumin (g/dL)	4.2 [3.6, 4.3]	4.0 [3.8, 4.1]	0.657
IBD duration (years)	3.05 [1.93, 5.21]	4.18 [1.14, 4.53]	0.775
IFX trough (μg/mL)	25.28 [5.35, 38.96]	15.54 [7.11, 24.15]	0.540
IFX dose (mg)	500 [500, 700]	500 [400, 600]	0.738
IFX dose (mg/kg)	9.24 [7.45, 10.04]	9.56 [8.49, 10.03]	0.838
IFX interval (weeks)	4.0 [4.0, 6.0]	5.0 [4.0, 6.0]	0.554
Days since IFX dose (days)	32.0 [28.0, 42.0]	35.0 [28.0, 42.0]	0.967
_adj_IFX dose (mg/kg/day)	0.30 [0.18, 0.33]	0.24 [0.22, 0.36]	0.967
_adj_IFX Trough (μg/mL per mg/kg/day IFX)	69.06 [36.56, 127.63]	55.03 [36.04, 66.78]	0.653
Weekly MTX dose (mg)	12.50 [10.00, 17.50]	10.00 [10.00, 17.50]	0.701
Weekly MTX dose (mg/kg)	0.20 [0.17, 0.23]	0.23 [0.15, 0.45]	0.653

## Data Availability

Data are not publicly archived, but deidentified data can be made available upon request.
